# Biocomposite-Based Biomimetic Plate for Alternative Fixation of Proximal Humerus Fractures

**DOI:** 10.3390/biomimetics10100688

**Published:** 2025-10-13

**Authors:** Miguel Suffo, Irene Fernández-Illescas, Ana María Simonet, Celia Pérez-Muñoz, Pablo Andrés-Cano

**Affiliations:** 1 Department of Mechanical Engineering and Industrial Design, Biomedical Research and Innovation Institute of Cádiz (INiBICA), High School of Engineering, University of Cadiz; Avda. Ana de Viya, 21, 11009 Cádiz, Spain; 2 Department of Orthopaedic Surgery and Traumatology, Puerta del Mar University Hospital, Biomedical Research and Innovation Institute of Cádiz (INiBICA), Avda. Ana de Viya, 21, 11009 Cádiz, Spain; irenefernandezillesca@gmail.com (I.F.-I.); pabloanca@gmail.com (P.A.-C.); 3 Department of Organic Chemistry, Institute of Biomolecules (INBIO), Faculty of Sciences, University of Cadiz, Agrifood Campus of International Excellence (ceiA3), Puerto Real, 11510 Cádiz, Spain; ana.simonet@uca.es; 4 Department of Biomedicine, Biotechnology and Public Health, Faculty of Medicine, University of Cadiz, Avda. Ana de Viya, 21, 11009 Cádiz, Spain; celia.perez@uca.es

**Keywords:** proximal humerus fractures, biocomposite implants, patient-specific implants, engineering-surgical collaboration, Sugarcane by-products (BCF), biocompatibility

## Abstract

Proximal humerus fractures are frequent injuries that often require internal fixation. Conventional metallic plates, however, present significant drawbacks such as corrosion, secondary removal surgeries, and adverse reactions in patients with metal hypersensitivity. This study evaluates biocomposite plates fabricated from polylactic acid (PLA) and polyvinyl alcohol (PVA), reinforced with hydroxyapatite (HA) derived from sugar industry by-products (BCF) at 10% and 20% concentrations. These composites are compatible with both injection molding and 3D printing, enabling the design of patient-specific implants. Characterization by SEM, FTIR, XRD, and DSC confirmed that BCF incorporation enhances strength, stiffness, osteoconductivity, and biocompatibility. Mechanical testing showed that PVA/BCF exhibited greater tensile strength and stiffness, suggesting suitability for load-bearing applications, though their water solubility restricts use in humid environments and prevents filament-based 3D printing. PLA/BCF composites demonstrated better processability, favorable mechanical performance, and compatibility with both manufacturing routes. Finite element analysis highlighted the importance of plate–humerus contact in stress distribution and fixation stability. Compared with non-biodegradable thermoplastics such as PEI and PEEK, PLA/BCF and PVA/BCF offer the additional advantage of controlled biodegradation, reducing the need for secondary surgeries. Cell viability assays confirmed cytocompatibility, with optimal outcomes at 10% BCF in PVA and 20% in PLA. These results position PLA/BCF and PVA/BCF as sustainable, patient-tailored alternatives to metallic implants, combining adequate mechanical support with bone regeneration potential.

## 1. Introduction

Due to factors such as population aging and the rise in sports-related trauma, shoulder injuries, particularly proximal humerus fractures, have become highly prevalent, representing approximately 5% of all fractures [1,2]. They constitute the second most common fracture type and the third most frequent in individuals over 65 years of age [3]. These fractures are particularly challenging to manage, as they often extend into the metaphyseal region [4]. Among the available treatment options, osteosynthesis with plates and screws remains the most widely employed approach [5]. According to Rischen et al. [5], successful bone healing requires not only appropriate biological conditions but also specific mechanical factors. Two distinct types of bone healing have been described [6]: osteonal and non-osteonal. Under rigid osteosynthesis, osteonal healing occurs without callus formation, provided that fracture fragments are in direct contact through the stability offered by fixation plates. This mechanical stabilization modulates the biomechanical environment at the fracture site, ensuring sufficient stability to prevent fragment displacement [7].

These implants must be manufactured from biocompatible materials, capable of interacting with human tissue without eliciting adverse effects and resistant to bacterial colonization. In addition, they are required to fulfill cytotoxicity and osteoinductive criteria [8]. Contemporary osteosynthesis systems consist of plates and screws available in multiple sizes and geometries, most commonly fabricated from stainless steel or titanium alloys [7,9]. A well-recognized limitation of metallic plates is their susceptibility to corrosion, which can induce toxic effects within the body [9,10]. The subsequent release of metal ions or organometallic particles may lead to both local and systemic toxicity, potentially triggering implant rejection [11]. Furthermore, implants are subjected to micromotion due to dynamic physiological loading and remain continuously exposed to body fluids such as blood, synovial fluid, and lymph. This combination, together with the direct interface between the plate and cortical bone, increases the risk of metallosis [12,13]. Additional drawbacks of metallic implants include loosening and stress shielding caused by the mismatch in elastic modulus between implant and bone tissue [7,14], which can lead to progressive humeral bone loss [10,15].

To overcome these limitations, alternative materials have been proposed to replace metals in bone implants, focusing on biocompatible options with favorable mechanical properties, such as rigid thermoplastics and bioceramics [16]. Polymeric materials and their composites offer the additional advantage of compatibility with diverse manufacturing techniques, including injection molding and additive manufacturing via fused filament fabrication (FFF) [17]. Injection molding enables the efficient production of standardized implants, whereas FFF allows the fabrication of patient-specific implants or plates tailored to the geometry of each fracture [18,19].

For successful healing of proximal humeral fractures, the ideal implant material should degrade gradually, thus transferring mechanical loads progressively from the implant to the bone [20]. Among the available polymers, polyvinyl alcohol (PVA) has been identified as a highly suitable candidate. PVA is a synthetic, biocompatible, biodegradable, non-toxic, and hydrophilic polymer [21,22]. Structurally, it is a semi-crystalline copolymer of vinyl alcohol and vinyl acetate, recognized for its high chemical resistance, viscoelastic behavior, and excellent mechanical performance, including strength, toughness, low friction, and lubricity [23]. Its viscoelastic properties are comparable to those of articular cartilage [22,24], and can be further enhanced through the incorporation of bioactive and osteoconductive additives such as bioceramics [14].

Another polymer of particular interest is polylactic acid (PLA), a hydrophobic aliphatic polyester that exhibits favorable mechanical properties, as well as high biocompatibility and biodegradability [25]. PLA is synthesized from lactic acid (LA) [26], a natural organic acid produced through the fermentation of sugars derived from renewable resources, which positions it as an environmentally friendly material. Nevertheless, its synthesis requires catalysts and strictly controlled conditions, such as temperature, pressure, and pH together with extended polymerization times, resulting in considerable energy consumption. A further advantage of PLA lies in its processing versatility, as it can be molded into various forms, including scaffolds, sutures, rods, films, nanoparticles, and micelles. Owing to its bioresorbability and biocompatibility, PLA has gained widespread use in biomedical applications, particularly in bone fixation devices [24].

Multiple studies have highlighted the versatility of PLA in biomedical applications [27]. For instance, Tyler et al. [26] reviewed recent advances in bone replacement structures manufactured using 3D printing technologies. PLA scaffolds have demonstrated suitable biocompatibility, supporting the viability and proliferation of osteosarcoma cells and exhibiting higher human osteoblast (hFOB) viability compared to titanium samples [28]. Moreover, PLA can be processed as a composite with bioactive inorganic fillers such as tricalcium phosphate (TCP) [29,30], bioactive glass [31], and particularly hydroxyapatite (HA) [32,33]. Matsuo et al. [34] reported enhanced bone healing in two patients with mandibular lesions when using PLLA reinforced with 50 wt% HA, compared to titanium. Importantly, both PLA and PVA have been approved by the U.S. Food and Drug Administration (FDA) for medical applications [35,36].

Ceramic materials are characterized by their high compressive strength and hardness, as well as by an elastic modulus that exceeds that of bone. Among them, calcium phosphate ceramics are widely applied as osteoconductive bone graft substitutes [8]. The most commonly used types are hydroxyapatite (HA) and tricalcium phosphate (TCP), owing to their high biocompatibility, bioactivity, osteoconductivity, and variable degradation rates [11]. Suffo et al. [37], described a procedure to obtain an HA-like biocomposite with proven cell viability as a bone substitute, demonstrating that one possible route for synthesizing HA involves the valorization of sugar industry by-products. Previous studies have reported that combining PVA with HA enhances cell adhesion and proliferation, indicating high bioactivity [23,24,25]. More recently, patent P202330826 [38], described the development of a biocomposite named BBCFiller^®^ (hereinafter BCF), consisting of nanohydroxyapatite, designed for use as a bone filler or as a surface coating to improve osteoinductivity. BCF exhibits properties comparable to HA, with excellent cell adhesion and proliferation performance. The evaluation of these materials depends not only on their intrinsic properties but also on the specific characteristics of the biological host. Hence, biocompatibility must be understood as a property of the overall system rather than of the material in isolation. Strictly speaking, there is no such thing as a universally biocompatible material. In this manuscript, the term “biocompatible” will therefore be used to denote an “intrinsically biocompatible system,” while retaining the simpler term for the sake of readability [39].

This study presents a comparative analysis of PLA and PVA polymers for the development of novel biocomposites incorporating HA. The objective is to identify the optimal formulation for an osteosynthesis plate specifically designed for the treatment of proximal humeral fractures. The originality of this work lies in the design and validation of a new polymer-based plate, proposed as a clinically relevant alternative to conventional metal implants. Unlike metallic systems, the proposed biocomposite aims to reduce complications associated with corrosion, ion release, and stress shielding, while promoting biological integration and gradual load transfer during fracture healing. To achieve this, two different polymeric matrices were assessed, each reinforced with two proportions of BCF, and their manufacturing feasibility was validated through both injection molding and additive manufacturing via FFF. This dual approach highlights not only the versatility of the proposed materials but also their potential to enable patient-specific implant designs, aligning with current trends in personalized orthopedics.

## 2. Materials and Methods

This study aims to develop and characterize biocomposites based on PVA and PLA polymers reinforced with hydroxyapatite-based fillers (BCF) for potential applications in bone repair and osteosynthesis. A comprehensive experimental methodology was designed to evaluate the physical, chemical, and mechanical properties of the base materials and their composites. Section 2 details the materials employed and the methods used for their preparation, characterization, and analysis, forming the basis for the results and discussion presented in subsequent sections.

### 2.1. Materials

Table 1 summarizes the key physical and mechanical properties of PVA and PLA filaments, as provided by the manufacturers Ultimaker B.V. (New York, NY, USA) and Smart Materials (Valencia, Spain), with a nominal diameter of 1.75 ± 0.10 mm. The main selection criterion was their bioabsorbable and biodegradable nature [25,40].

BCF was synthesized following the method reported by Suffo et al. [37], whose basic characteristics are summarized in Table 2.

### 2.2. Fabrication of PVA/BCF and PLA/BCF Composites

**Figure 1:**
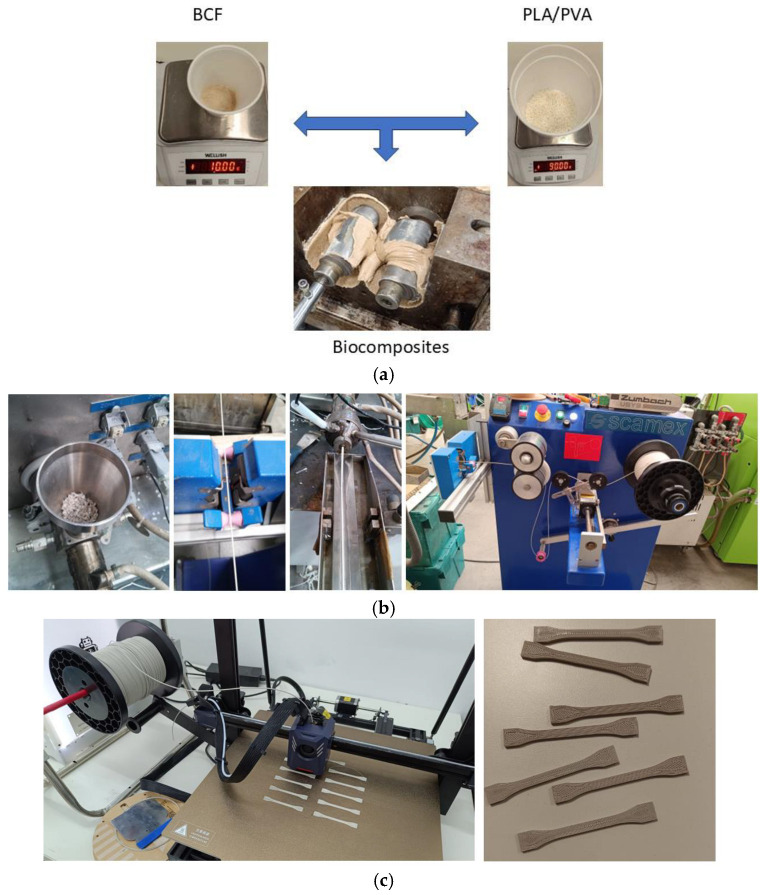
Manufacturing processes of the composites, 3D filament, and 3D-FFF printed samples: (**a**) Physical appearance of dry BCF and PVA/PLA prior to mixing, alongside the PVA/PLA blended with BCF inside the internal mixer; (**b**) Fabrication of the PLA10 3D filament, showing the feeding hopper for the PLA10 composite, extrusion of the molten filament, water-cooling chamber, and the final filament winding spool; (**c**) 3D-printed specimens produced using the PLA10 composite material.

The mixing parameters were selected based on previous studies of similar biocomposites [16]. The constituents were combined using a melt mixing technique, a method widely employed for comparable materials. This approach has been validated in prior work involving thermoplastic matrices processed with the same equipment [41], ensuring homogeneity and reproducibility of the blend. Following the protocol described in [16], the melt mixing was performed using a Scamex Rehoscam internal mixer (France), operating with a small input volume to optimize subsequent extrusion and injection molding processes for the PVA and PLA-based biocomposites. Figure 1 shows the appearance of BCF and PLA/PVA materials before and after mixing.

Prior to mixing, the materials were oven-dried at 60 °C for 24 h. Subsequently, a series of tests were performed to optimize the mixing process for PVA and PLA composites loaded with 10 wt% and 20 wt% BCF [16], resulting in the following optimal parameters: a temperature range of 183–185 °C, a screw speed of 60 rpm, and a mixing time of 7–8 min. Virgin polymers were processed under the same conditions as the biocomposites. BCF was initially ground using a milling device and then sieved to obtain a particle size of 0.1 mm. During the mixing process, the temperature of the mixer chamber was first raised to 155–175 °C for the PVA and PLA composites (see Table 3), at which point the polymer was introduced. The rotation speed was allowed to stabilize at 60 rpm. Once the chamber temperature reached a steady state between 165 and 185 °C, the ground and dried BCF was added to the mixture.

Figure 1b illustrates the fabrication of the PLA10 3D printing filament under controlled conditions of 148 °C temperature, 8 bar pressure, and a screw speed of 25 rpm, consistently maintaining a filament diameter of 1.72 ± 0.04 mm. The figure comprehensively depicts key stages of the process, including the feeding hopper for the PLA10 composite material, extrusion of the molten filament, water-cooling chamber for rapid solidification, and collection of the filament onto the winding spool.

The biocomposites were processed using a WSGM-250 mill (J. Purchades, Ibi, Spain), specifically designed for cutting polymer-based materials. This device is equipped with stainless steel blades capable of reducing the material to particle sizes suitable for subsequent injection molding. After milling, the fragments were sieved to ensure a minimum particle size of 3 mm, considered optimal for the injection process [42]. Prototype fabrication was performed using the highest feasible loading of BCF, based on the processing behavior and mass distribution of the PVAX and PLAX blends, where X denotes the weight percentage of BCF incorporated, and the remainder corresponds to the polymer matrix.

### 2.3. Chemical Characterization

The distribution of BCF particles within the polymeric matrix and the morphological features of the PVA/PLA biocomposites were examined using a scanning electron microscope (SEM) (S4800, Hitachi, Tokyo, Japan) equipped with an energy-dispersive X-ray spectroscopic detector (EDX). Specimens were coated with a thin gold layer using a sputter coater (108auto, Cressington, Watford, UK) to ensure electrical conductivity and surface stability. Micrographs were acquired at magnifications of 500× and 8000×, employing an electron landing energy of 10 kV. These imaging parameters were optimized to provide high-resolution surface morphology while minimizing sample damage. Functional groups of the filaments were characterized by Fourier Transform Infrared Spectroscopy with Attenuated Total Reflectance (FTIR-ATR) (UMA-600 Microscope, Varian Excalibur Series). Spectral acquisition was performed using a standard resolution of 4 cm^−1^ within the range of 4000–400 cm^−1^ and 64 accumulations per sample. X-ray Diffraction (XRD) analysis was conducted with monochromated Cu Kα radiation (Rigaku Ultima III, Woodlands, TX, USA) to evaluate the crystallographic structures of BCF, PLA, PVA, and the biocomposites. Operating conditions were 40 kV voltage and 44 mA current, with a 2θ scanning range from 10° to 45°, a step size of 0.04°, and a scan speed of 1°/min. Diffraction peaks of BCF were identified by comparison with the ICDD reference pattern 09-0432.

Energy-dispersive X-ray spectroscopy (EDS) mapping was performed in conjunction with SEM imaging to analyze the spatial distribution of elements in BCF, PLA, PVA, and their composites. Measurements were conducted using an EDS detector integrated into the SEM system. Elemental distribution maps for calcium (Ca), phosphorus (P), and oxygen (O) were generated to confirm the homogeneous dispersion of BCF particles within the polymer matrices.

### 2.4. Mechanical, Thermal and Rheological Test

The thermal properties of the biocomposites and pure polymers were analyzed using Differential Scanning Calorimetry (DSC). The degree of crystallinity (χc) was calculated according to Equation (1), following the methodology detailed in [16,43]:

(1) $$\chi_{c}=\frac{H_{c(polymer)}}{H_{Biocomp}\cdot w_{polymer}}$$

where

Hc_polymer_ is the enthalpy of crystallinity of the pure polymer at 100% crystallization;

Hc_Biocomp_ is the enthalpy of crystallinity of the biocomposite; and

W_polymer_ is the percentage of mass polymer in the biocomposite (1, 0.9, 0.8).

Hc_PLA_ is the melt enthalpy of 100% crystalline PLA and is equal to 91 J/g [44,45], and Hc_PVA_ is equal to 156 J/g [46].

Differential Scanning Calorimetry (DSC) analyses were performed independently on pure polymers and biocomposites using a Mettler Toledo 1/200 DSC system (Columbus, OH, USA), equipped with a cryogenic module capable of operating between −37 °C and 200 °C. The system allows controlled nitrogen gas flow to maintain stable thermal conditions during measurements.

Approximately 13.6 mg of each sample was tested, in a nitrogen atmosphere with a flow rate of 50 mL/min. The procedure complies with UNE-EN-ISO 11357 standards [47], employing 40 μL aluminum crucibles. The thermal program consisted of five steps:

1. Isothermal hold at 25 °C for 5 min
2. Heating from 25 °C to 220 °C at 10 °C/min
3. Cooling from 220 °C to 25 °C at −10 °C/min
4. Isothermal hold at 25 °C for 5 min
5. Reheating from 25 °C to 220 °C at 10 °C/min

Flowability measurements were performed to evaluate the influence of residual material on the rheological behavior of the polymer composites. The melt flow index (MFI) was determined using an MP600 extrusion plastometer (Tinius Olsen, Oslo, Norway) according to ISO 1133-1:2012 standards [48]. Samples weighing 5 g and 7 g of chopped material were tested under a 2.16 kg load at an extrusion cylinder temperature of 230 °C, with a shear time interval of 5 s. The initial phase corresponds to material homogenization, followed by a transition that indicates the polymer melting point (Tm). The subsequent feature observed in all curves corresponds to the pulse line or solidification. For ease of comparison, the durations of these phases were shortened in the graphs [41].

PLA, PVA, and biocomposite formulations were processed using an Engel Victory 28 injection molding machine (Engel Group, Schwertberg, Austria) to produce standardized specimens for mechanical testing. Following previously established methodologies [16], t mechanical behavior was evaluated using specimens designed for each formulation in accordance with ISO 527-1:2012 standard [49], recommendations for tensile testing. For comparative purposes, an additional set of PLA10 composite specimens was fabricated by 3D printing using an Anycubic Kobra 2 Max printer. Printing parameters included a nozzle temperature of 148 °C, a layer height of 0.2 mm, and a build plate temperature of 60 °C. A nozzle diameter of 0.4 mm was used, and specimens were printed with 100% infill. Printing was performed in the X–Y orientation, which was selected as the critical configuration based on [9], as it represents the orientation expected to exhibit the lowest mechanical performance (see Figure 1c).

For each specimen, Young’s modulus (E), maximum tensile strength (σ_max_), and elongation at break (ε) were determined through tensile testing under standardized conditions. These parameters provide a comprehensive understanding of the material’s elastic response, failure resistance, and deformation capacity, enabling an in-depth evaluation of the composite’s mechanical performance.

Impact resistance was evaluated according to the ISO 179 standard [50], using a Charpy-Izod IMPATS 15 pendulum tester (Metrotec, San Sebastián, Spain). For each formulation, five to ten specimens measuring 80 mm × 10 mm × 2 mm were tested, as specified by the standard. Tests were conducted with pendulum energies ranging from 2 J to 5 J and an impact velocity of 2.9 m/s. Additionally, tensile strength characterization was performed using a Tinius Olsen H10KS Universal Testing Machine in compliance with UNE-EN ISO 527-1 and ISO 527-2 standards [51]. The system employs interchangeable load cells with capacities of 100 N and 10 kN, depending on the expected force range of the samples. It features a maximum grip separation of 1100 mm to accommodate various specimen sizes. Data acquisition was conducted at a nominal rate of 200 Hz to ensure accurate recording of load and displacement data. The machine is equipped with a high-precision displacement measurement system capable of resolutions down to 0.001 mm. All tests were carried out at a controlled crosshead speed of 0.5 mm/s. Specimens were prepared according to standardized dimensions specified in the relevant ISO norms to ensure reproducibility and comparability of results.

### 2.5. Cell Viability Assay

The viability test evaluated human fetal osteoblastic cells (hFOB 1.19) exposed to PVA, PLA, and BCF biocomposites for osteosynthesis applications, using MTS assays at 24, 48, 72 h, and 7 days. Cells were cultured in osteogenic media (DMEM with 10% FBS and antibiotics) at 35 °C and 5% CO_2_, seeded in 96-well plates (2 × 10^4^ cells/well), and treated with sterilized (120 °C for 20 min) material samples at concentrations of 15–100 mg/mL in conditioned medium (DMEM + 1% FBS). Controls included untreated cells (positive control, C+) and methanol-fixed cells (negative control, C−). After incubation with test solutions at 34 °C and 5% CO_2_, MTS reagent was added, followed by a 1 h incubation and absorbance measurement at 490 nm using a Varioskan LUX reader. Experiments were performed in triplicate across three independent replicates to ensure reproducibility and robust assessment of biocompatibility over critical time points.

### 2.6. Segmentation of the Proximal Humerus

**Figure 2:**
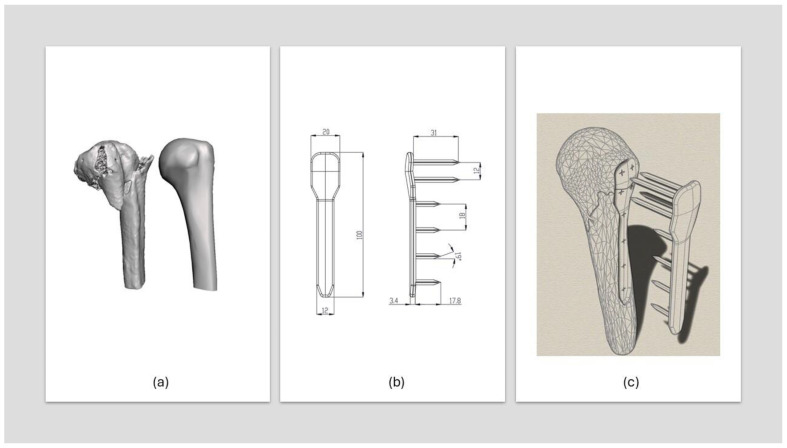
Three-dimensional reconstruction of the fractured humerus and assembly: (**a**) Fractured humerus model and healthy humerus model; (**b**) Plate measurements; (**c**) Assembly of the components.

To ensure an accurate anatomical fit in the osteosynthesis plate design, the proximal humerus was segmented using 3D Slicer 5.8.1. software from DICOM images of a fractured humerus. The CT sequence with the thinnest slices was selected to maximize geometric precision. Due to the absence of CT images of the patient’s contralateral (right) humerus, a generic 3D model of the proximal humerus titled “Humerus_Right Human Skeleton Upper Arm Bone” (creator: profguy, Thingiverse, published 14 June 2014) was obtained from Thingiverse. This model (Figure 2a), released under Thingiverse licensing terms, served as the reference geometry for the custom plate design.

### 2.7. Osteosynthesis Plate Design

**Figure 3:**
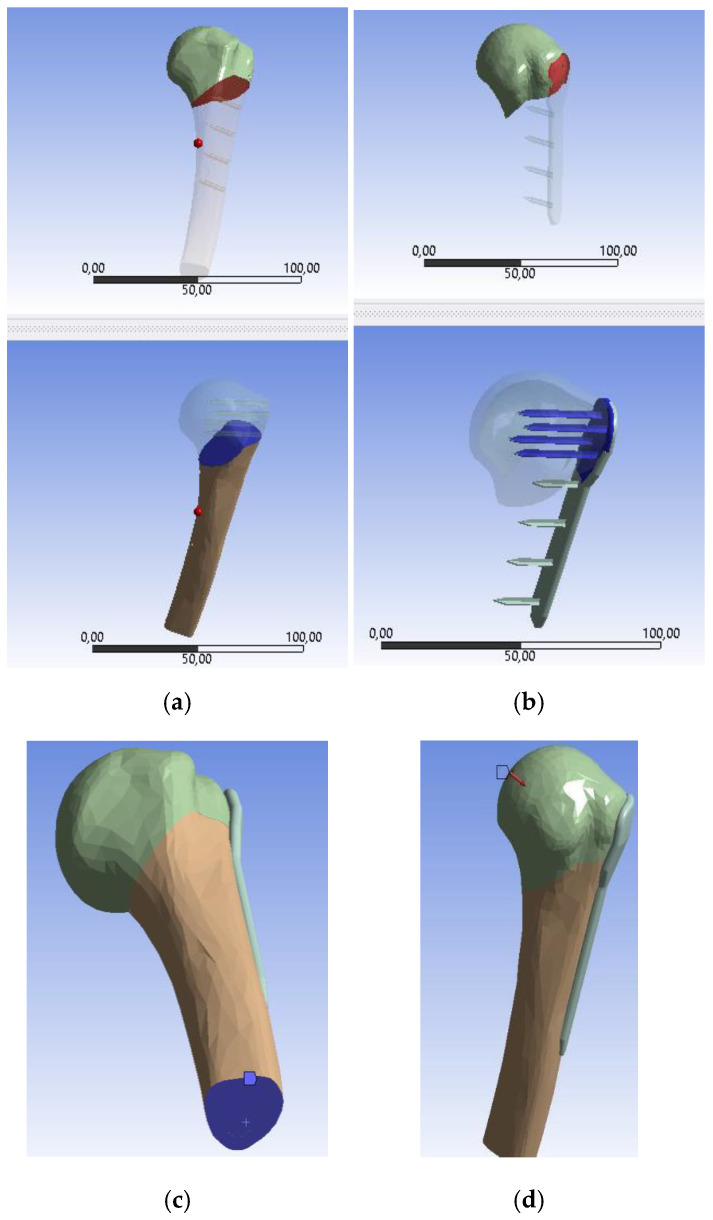
Boundary conditions for the simulation; (**a**) Frictional contact areas; (**b**) Bonded-type areas; (**c**) Fixation of the distal end of the bone; (**d**) Applied force on the humeral head.

The osteosynthesis plate was designed using Autodesk Fusion 360 2025 (educational license) CAD software to anatomically conform to the bone’s morphology and meet functional requirements, with placement typically on the lateral side near the lesser tuberosity. Prior to fixation, fracture reduction is performed using a healthy humerus model to ensure anatomical restoration. Unlike conventional metallic plates, which pose corrosion risks, this design employs a polymer-bioceramic composite (PVA/PLA with BCF), eliminating the need for metallic screws by incorporating spike-shaped anchors for stabilization. The plate’s contact surfaces are coated with a biocompatible adhesive [44]. Both cancellous and cortical bone regions were modeled, with the cortical bone assigned a thickness of 3.5 mm. A 54° osteotomy simulates the humeral fracture, dividing the bone into two segments, and bone milling is modeled by material removal to ensure a precise, patient-specific fit, enhancing stability while minimizing localized pressure. Figure 3b,c illustrate the humerus-plate assembly, milled area, and plate geometry measurements.

### 2.8. Finite Element Method (FEM) Simulation

The bone-plate assembly was modeled in CAD software, resulting in five solid components: superior cortical bone, inferior cortical bone, cancellous bone, and the fixation plate. These components were subsequently imported into the FEM simulation environment (ANSYS Academic Research, ANSYS Inc., Houston, PA, USA). Static analysis was carried out by incorporating new materials into the ANSYS Workbench 2021 R1 library. PLA10 was selected as the plate material due to its favorable biomechanical and rheological properties, previously reported in the literature, as well as its compatibility with fused deposition modeling. This selection also accounted for the tendency of PVA-based biocomposites to disintegrate during post-extrusion water-bath drying because of their high water solubility. The mechanical properties of cortical and cancellous bone were assigned from published data, with all simulation materials summarized in Table 3.

**Table 3:** Mechanical properties of the materials incorporated in ANSYS for the FEM simulation.

	Cortical Bone [45]	Cancellous Bone [14]	PLA10
Density [kg/m^3^]	2000	1180	1240
Young’s Modulus [GPa]	15	1.18	2.71
Poisson’s Ratio	0.3	0.39	0.3
Tensile Yield Strength [MPa]	114	15	54.2
Tensile Ultimate Strength [MPa]	133	20	59.37

The fracture-line bone surfaces were modeled as frictional contacts, with a coefficient of friction of 0.3 [46] (Figure 3a), bonded contacts [45,46,52] (Figure 3b) were applied to the bone-plate interfaces, representing rigidly joined surfaces without sliding or separation, in a manner analogous to welding. This configuration was adopted since sliding behavior was not considered critical for the present analysis.

To evaluate mesh dependency, an absolute error analysis was performed across different iterative cases by varying the element size while keeping all other parameters constant. The ASME Grid Convergence Index (GCI) method [14,53] was applied, with element sizes in the ranges 0.4–0.5 mm and 0.5–0.55 mm. A maximum allowable GCI error of 5% was satisfied using an element size of 0.5 mm, yielding a total of 447,412 tetrahedral elements. The boundary conditions were defined by fixing the base of the humerus, including both cortical and cancellous regions [54] (Figure 3c). Following the methodology described in [52], the load borne by the proximal humerus during a 90° abduction was simulated, corresponding to a maximum compressive load equivalent to 80% of body weight applied to the humeral head. As illustrated in Figure 3d, the bone is oriented at an angle of approximately 50°, and a force of 640 N was applied to the humeral head, representing an individual weighing 80 kg. Under these conditions, the simulation aimed to evaluate plate stability under load using Ansys Mechanical APDL (static structural analysis). Additionally, the efficiency of the plate–bone interface was assessed, following an approach analogous to that used by Suffo et al. [14] in simulating of hip prosthesis components.

## 3. Results and Discussion

### 3.1. Compositional Analyses

**Figure 4:**
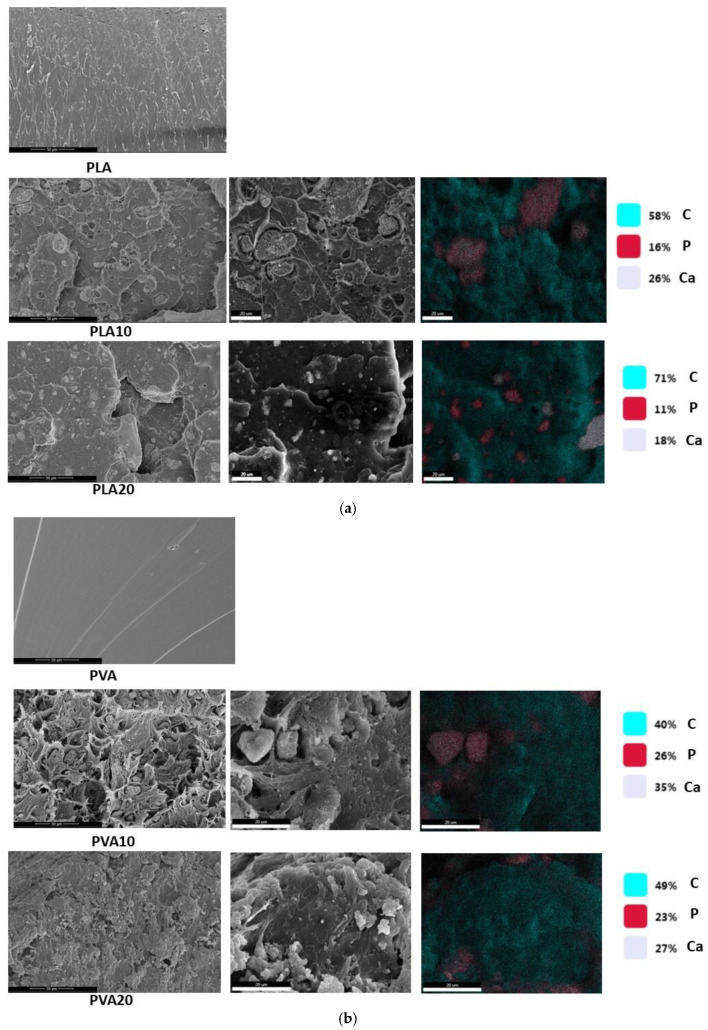
SEM analyses of PLA- and PVA-based biocomposites. (**a**) Micrographs of pure PLA and PVA, along with PLA10 and PVA10 composites, acquired at different magnifications. Corresponding energy-dispersive X-ray spectroscopy (EDX) elemental maps are provided, displaying the distribution of carbon (C, blue), phosphorus (P, red), and calcium (Ca, grey). (**b**) Micrographs of PLA20 and PVA20 composites at different magnifications, with corresponding EDX maps illustrating the elemental distribution of C (blue), P (red), and Ca (grey).

The physicochemical characterization of PVA/PLA samples and their biocomposites was performed using SEM, as shown in Figure 4. The images are organized by polymer type and presented with increasing BCF content, while higher magnifications highlight finer microstructural features. SEM micrographs of neat PLA and PVA display relatively smooth and homogeneous surfaces at low magnification, which evolve into more fibrous or laminar patterns at higher resolution. Both polymers exhibit micrometer-scale surface roughness. Figure 4a shows SEM images of PLA composites containing 10% and 20% BCF at different magnifications. The micrographs confirm the incorporation of BCF particles in the PLA matrix. Compared to neat PLA, the composites exhibit more irregular and less homogeneous morphologies, with a distinctly granular surface texture attributed to BCF addition. The particles are embedded within the polymer, although their distribution is not completely uniform. At higher magnifications, inclusions ranging from the nanometer to micrometer scale can be identified, indicating heterogeneous dispersion. The incorporation of BCF considerably increases surface roughness, which may enhance cell adhesion. Furthermore, the presence of hydroxyapatite (HA) and tricalcium phosphate (TCP), the main mineral constituents of BCF, substantially modifies PLA morphology, producing rougher, more heterogeneous, and porous structures containing micrometer-scale particles. These findings are consistent with previously reported effects of mineral fillers on polymer matrices. Figure 4b presents SEM images of PVA composites, in which the increase in surface roughness is even more pronounced than in PLA-based systems, becoming clearly visible across the sample surfaces.

EDX (Energy-Dispersive X-ray Spectroscopy) analyses further confirmed the elemental composition of the biocomposites, verifying the presence and distribution of BCF-associated elements. The spectra indicated a predominance of carbon (C), followed by calcium (Ca). The detection of both calcium and phosphorus (P) confirmed the successful incorporation of BCF, yielding a Ca/P ratio of 1.63, which is very close to that of stoichiometric hydroxyapatite (1.67). The high carbon content, consistent with expectations, originated from the polymeric matrices.

In summary, SEM analyses revealed marked morphological and microstructural differences between the neat polymers and their BCF-containing composites, with PVA-based systems exhibiting more pronounced changes than their PLA counterparts. The incorporation of BCF increased surface roughness and heterogeneity and resulted in a non-uniform elemental distribution, as further substantiated by elemental mapping.

**Figure 5:**
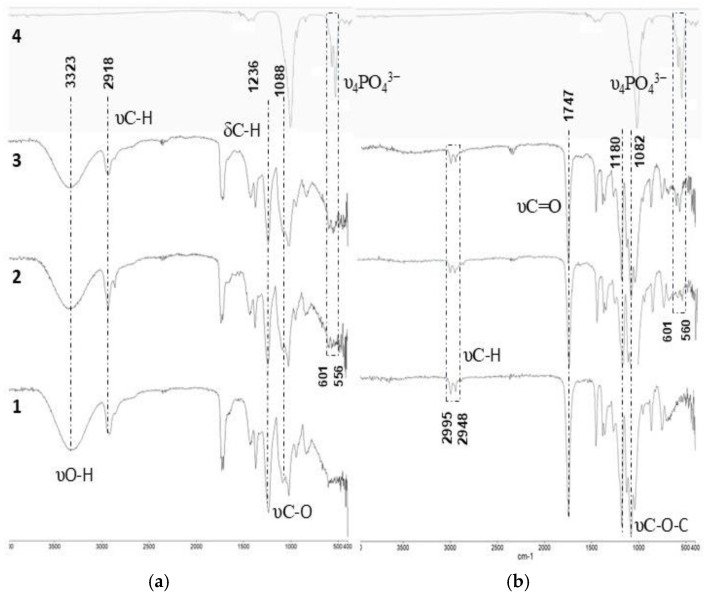
FTIR analyses of pure polymers, BCF, and their biocomposites. (**a**) Spectra of pure PVA (row 1), PVA10 (row 2), PVA20 (row 3), and pure BCF (row 4). (**b**) Spectra of pure PLA (row 1), PLA10 (row 2), PLA20 (row 3), and pure BCF (row 4).

Figure 5 presents the FTIR spectra of pure PVA, PLA, and BCF, along with those of polymer composites containing 10% and 20% BCF. The spectra exhibit characteristic absorption bands corresponding to both the polymer matrices and hydroxyapatite (HA) [21]. The FTIR spectrum of pure PVA spans the infrared region from approximately 3500 to 400 cm^−1^. Prominent peaks are observed at 2918 and 1236 cm^−1^, attributed to C–H stretching and bending vibrations of the carbon backbone, respectively. A broad absorption band centered around 3323 cm^−1^ and a peak at 1088 cm^−1^ correspond to O–H and C–O stretching vibrations associated with hydroxyl groups. The carbonyl (C=O) band at 1731 cm^−1^ is assigned to residual acetate groups remaining after the hydrolysis of polyvinyl acetate during PVA production [54].

For pure PLA, the FTIR spectrum displays the C=O stretching band at 1747 cm^−1^ [55], together with two C–O–C stretching bands at 1180 and 1082 cm^−1^ associated with the ester group. Characteristic C–H stretching vibrations of methyl and methine groups are observed at 2995 and 2948 cm^−1^, respectively. The FTIR spectrum of pure BCF exhibits typical PO_4_^3−^ vibrational bands, with prominent peaks at 1027, 601, and 560 cm^−1^ [37]. Since the 1027 cm^−1^ band of BCF overlaps with the C–O stretching bands of both PVA and PLA, particular attention was directed to the spectral region between 600 and 500 cm^−1^ to analyze the υ_4_ bands of PO_4_^3−^ ions, especially in composites containing 20% BCF.

**Figure 6:**
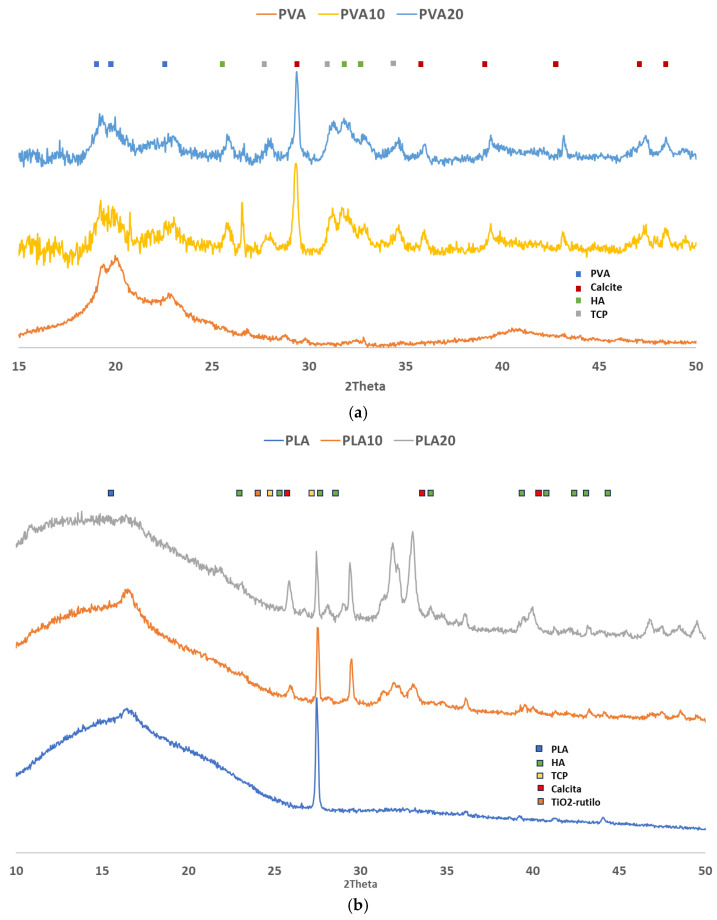
XRD patterns of pure polymers and their composites. (**a**) Pure PVA (orange), PVA10 (yellow), and PVA20 (blue). (**b**) Pure PLA (blue), PLA10 (orange), and PLA20 (grey).

X-ray diffraction (XRD) studies were performed to determine the crystalline phases of calcium phosphates, which require prior synthesis of the compound. The mechanical properties and bioactivity of the material are strongly dependent on the phase composition of the mineral component. Figure 6 shows the XRD patterns of pure PVA and its composites (PVA10 and PVA20). The X-axis represents the diffraction angle in degrees (2θ), defined as the angle between the incident X-ray beam and the diffracted beam, while the Y-axis corresponds to the intensity in counts per second (cps), reflecting the number of photons detected by the instrument per unit time.

In the PVA/BCF composite materials, the diffraction patterns of both compositions are similar, showing characteristic peaks of calcite, hydroxyapatite (HA), and tricalcium phosphate (TCP). These sharp and prominent peaks demonstrate the crystalline nature imparted by HA to the polymer matrix [56]. TPVA peaks detected around 2θ = 20° correspond to its crystalline phase, with crystal lattice parameters a = 1; b = 0; c = 1 [21]. The intensity and diffraction area of PVA reflections decrease significantly with increasing BCF content, suggesting that HA may partially hinder the ordering of PVA chains through particle confinement effects [57]. Analysis of the PLA-based samples indicates a predominantly amorphous structure. A reflection around 16.8°, superimposed on a broad diffuse band, is consistent with a largely non-crystalline organization of PLA. Notably, even in the pure PLA sample, intense reflections corresponding to TiO_2_ with a rutile crystalline structure are detected. The presence of this TiO_2_ phase, although initially unexpected given the reported origin of the PLA used, reveals the incorporation of rutile as a whitening agent during polymer processing. This observation provides insight into the manufacturing process of PLA and accounts for the detection of TiO_2_ in these samples [58].

### 3.2. Mechanical, Thermal, and Rheological Tests

**Figure 7:**
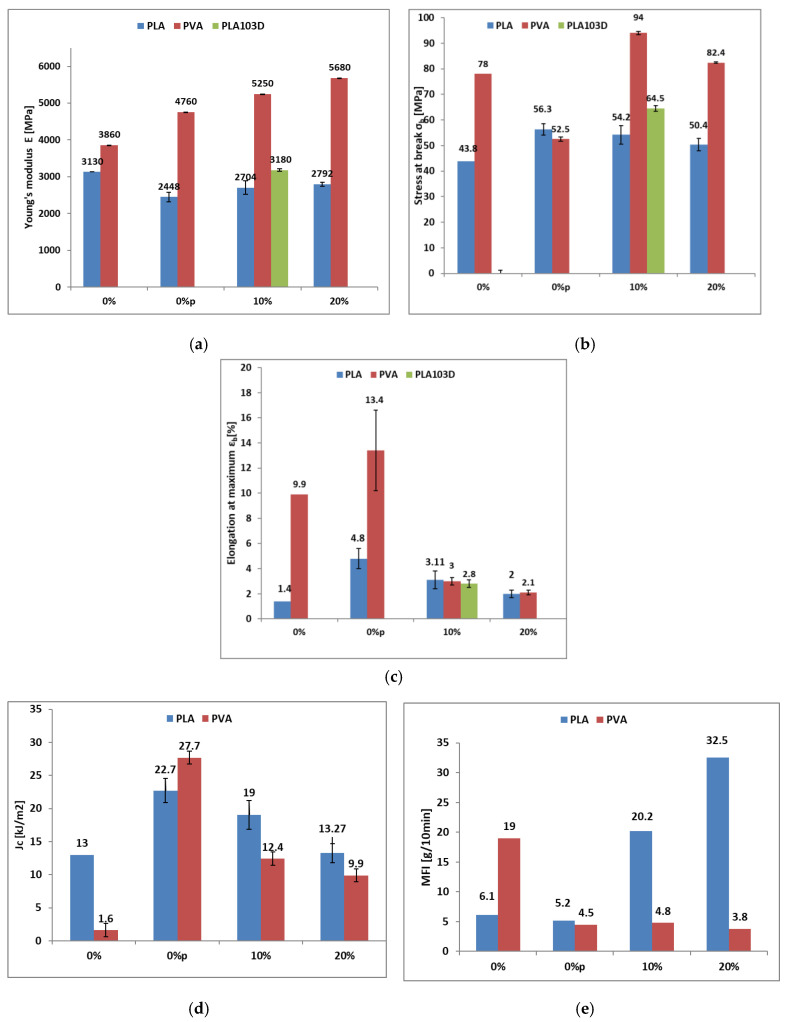
Mechanical properties of the biocomposites. (**a**) Young’s modulus (E); (**b**) Stress at break (σ_b_); (**c**) Elongation at maximum strain (ε_b_); (**d**) Impact strength (J_c_); (**e**) Melt flow index (MFI).

Figure 7a presents the tensile test results, highlighting the distinct mechanical behavior of the biocomposites as a function of their polymer/BCF ratio. This effect is particularly evident in the 3D-printed sample (PLA103D), whose stiffness exceeds that of pure PLA. For PLA-based composites, increasing the BCF content does not significantly affect the elastic modulus (Young’s modulus), indicating that rigidity remains essentially unchanged and that the additive does not provide a reinforcing effect. In contrast, PVA-based composites exhibit a clear increase in elastic modulus with increasing BCF content, consistent with the reinforcing or filler effect of the mineral phase [59]. Thus, despite the reduction in crystallinity revealed by DSC analysis, the stiffening effect imparted by BCF dominates. It is important to note that 0% corresponds to the pure polymer without mixer processing, whereas 0%p denotes the pure polymer after mixer processing, i.e., following one thermal cycle.

Figure 7b,c illustrate a similar trend in stress values: the 3D-printed specimen continues the pattern already observed in stiffness, with the composite material sustaining higher loads than any other sample tested. This observation justifies the FEM analysis performed using the properties of the injected sample (PLA10). In contrast, elongation decreases as the BCF content increases, consistent with previous reports on composites containing mineral-based fillers or additives [16]. This behavior confirms the reinforcing effect imparted by BCF [60]. The recorded values for the pure polymers are consistent with those presented in Table 1, which can be attributed to the thermal treatments applied during extrusion and injection processing. This phenomenon can be further explained based on the microstructural features identified in the biocomposites. SEM analyses showed interconnected fibrillated polymer structures formed by BCF incorporation, together with small voids and cavities. At the microscale, these fibrils and voids act to resist substantial deformation before failure, thereby enhancing ductility and limiting crack propagation. Therefore, the stability of the elastic modulus across varying BCF contents highlights the reinforcing role of solid by-products/wastes within the polymer matrix, in agreement with previously published findings [43].

The datasets (*n* = 14) were analyzed by parametric one-way ANOVA, yielding a significant Snedecor F value (F = 8.77) at a significance level of *p* < 0.05. The results indicate a significant effect of increasing the BCF percentage on the response variables for PLA, including Young’s modulus (*p* < 0.05, F = 5.61), strain at break (*p* < 0.05, F = 24.52), and elongation (*p* < 0.05, F = 5.61). For PVA, significant effects were also observed in Young’s modulus (*p* < 0.05, F = 7.00), strain at break (*p* < 0.05, F = 118.47), and elongation (*p* < 0.05, F = 45.07).

According to [60], PLA stress at break exhibited a slight decrease with the addition of BCF, reflecting the material’s stability under load, consistent with the behavior observed in the elastic modulus. In contrast, for PVA (Figure 7b), BCF incorporation led to increases in stress at break of 44% and 30% for composites with 10 wt.% and 20 wt.% filler, respectively, compared to the processed polymers. This suggests a degree of instability under load, similar to that observed in the elastic modulus.

Figure 7c shows a sharp decrease in elongation with increasing filler content, consistent with increased brittleness, a characteristic behavior in mineral-filled composites [61]. Although both materials are biocomposites, PLA maintains elongation values close to those of the pure polymer, whereas PVA is more sensitive to filler content.

The impact fracture energy per unit area (Jc) was evaluated using Charpy impact tests (Figure 7d). Consistent with [43], initially processed virgin PVA and PLA exhibited similarly high impact resistance, which gradually decreased with increasing plant-based filler content until stabilizing at approximately 20% filler. The reduction in impact resistance is attributed to polymer degradation via chain scission caused by exposure to two thermal cycles [60,62]. Despite the heterogeneous nature of the biocomposites, their mechanical behavior indicates good interfacial integration. However, as with mineral-based fillers, exceeding a certain filler concentration may lead to particle agglomeration, increasing stress concentrators, and thereby enhancing brittleness. This phenomenon can cause rapid crack propagation and fracture failure, but it was not observed for biocomposites with BCF content below 20%.

Similarly, ANOVA analysis within each dataset (*n* = 29) produced a significant Snedecor F value (*p* < 0.05, F = 66.66), indicating that time groups significant effect of increasing the BCF percentage on the response variable for PLA (*p* < 0.05, F = 55.09) and PVA.

As reported in [63], the melt flow index (MFI) of PLA biocomposites increases with BCF content (Figure 7e), suggesting that mineral fillers have an opposing effect compared to plant-based fillers, which decrease MFI as their concentration increases in the PLA matrix [59]. The flow behavior of the 10% PLA blend is similar to that of pure PVA, indicating good suitability for 3D filament production via additive manufacturing. Conversely, increasing BCF content in PVA composites reduces fluidity, indicating that particle addition hinders flow and increases mixture viscosity [46].

In PLA, the addition of 20% BCF increases fluidity by a factor of five, whereas the opposite trend is observed in PVA. These behaviors are characteristic of the semicrystalline nature of PLA and PVA, suggesting that the most degraded polymer chains initially belong to the amorphous phase [64]. One-way ANOVA analysis within each dataset (*n* = 17) revealed a significant Snedecor F value (*p* < 0.05, F = 1362.09) for the effect of increasing the BCF percentage on the MFI of PLA, and similarly significant results for PVA (*p* < 0.05, F = 51803).

**Figure 8:**
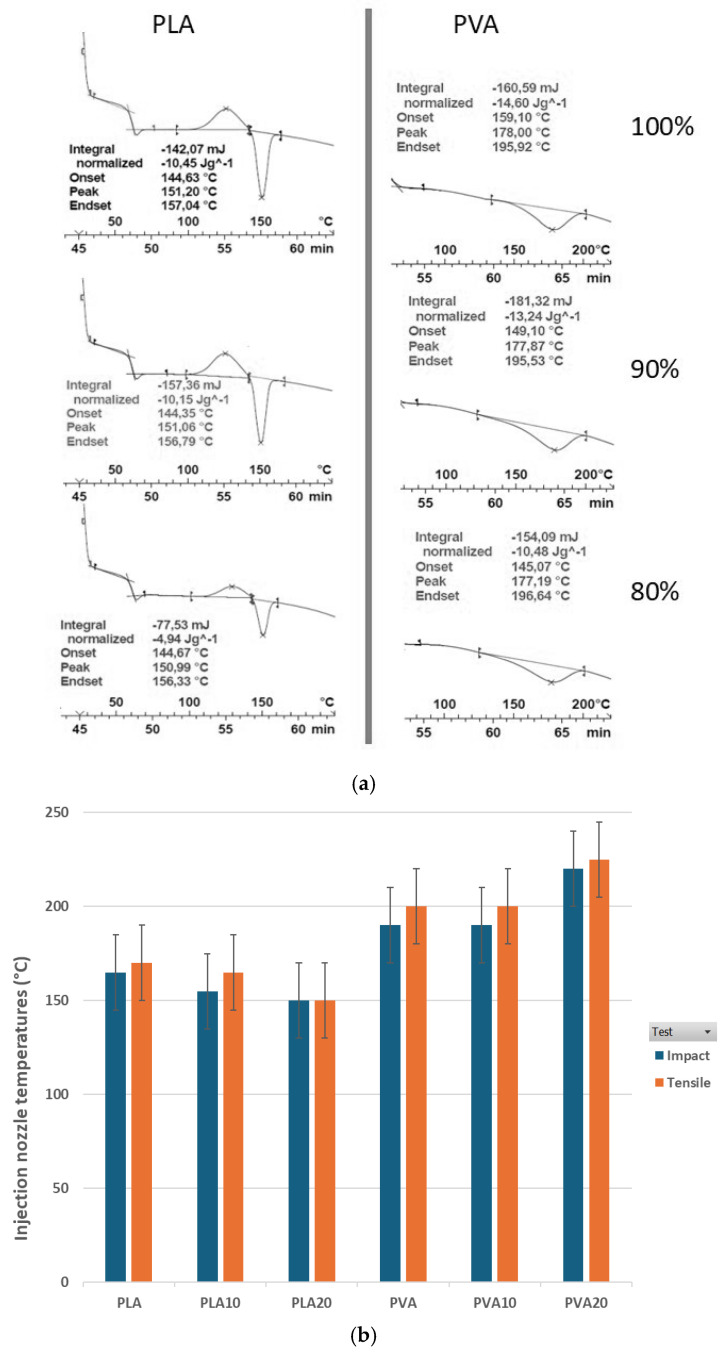
(**a**) DSC thermograms of pure PLA and PLA composites containing 10% and 20% BCF (left panel), and pure PVA and PVA composites with 10% and 20% BCF (right panel). (**b**) Injection nozzle temperatures used for test specimens (tensile and impact tests) corresponding to mixing ratios denoted as PVAX and PLAX.

The striped areas corresponding to pure PLA, PVA, and their biocomposites show remarkable similarities. The initial phase represents material homogenization, followed by a phase change corresponding to polymer melting (Tm). The subsequent line observed in all graphs is the pulse or solidification line. The enthalpy change (ΔH) and crystallization behavior remain consistent across samples. The melting temperature (Tm) of processed pure PLA was approximately 151.20 °C, while biocomposites exhibited Tm values between 150.99 and 151.06 °C, indicating no significant effect from BCF addition [45]. Figure 8b presents the injection nozzle temperatures used during specimen preparation for tensile and impact tests. In semicrystalline thermoplastics, polymer chains form irregular entangled coils in the molten state and reorganize into crystalline structures upon solidification [34]. According to [37], BCF acts as a nucleating agent in PLA, raising the crystallization temperature. Table 4 summarizes the crystallization rates of BCF-containing blends calculated using Equation (1). The crystallization rate of the 10:90 blend (χc) is not significantly affected by increased additive content, even without compatibilizing agents such as maleic anhydride (MAH) [65].

According to [66], the observed slight increase in crystallization rate (χc) in the blend may be attributed to the thermal processes undergone by the polymer during blending and extrusion of compound granules. However, a key difference from this reference is that the cited authors only incorporated up to 2.5% montmorillonite, whereas in the present study, higher additive contents were investigated.

The 20:80 blend exhibits a reduction in crystallinity χc by approximately half compared to the previous blend [16], which may explain the differences observed in the rheological behavior of the two biocomposites presented in Figure 8. This slight trend variation could be attributed to differences in particle size within the BCF. However, this effect does not appear to significantly influence the mechanical properties of either biocomposite. These observations suggest that BCF is a suitable additive that does not markedly alter the crystalline characteristics of the polymer matrix. According to [46], a similar phenomenon occurs in PVA-based mixtures, where the T_m_ is higher than that of PLA, indicating that more energy is required to melt PVA to achieve comparable crystallinity. An effect on crystallization behavior is evident when comparing PLA and PVA in the 20% BCF mixtures, with a noticeably diminished peak in PLA. As previously reported by Greco et al. 1987 [67], the crystallization behavior of semicrystalline polymers critically influences their mechanical properties, and the addition of secondary phases can affect the crystallinity and morphology of the polymer matrix.

Based on the values presented in Table 4, optimal injection temperatures for specimens used in mechanical testing were established. As illustrated in Figure 8b, PLA and its biocomposites were injected at temperatures approximately 15% lower than those used for PVA-based materials, translating into equivalent energy savings. This finding indicates that the injection molding process is more sustainable and produces a lower environmental impact compared to emerging manufacturing technologies, such as 3D printing, where the same materials are processed at temperatures around 190–220 °C [30,68].

Finally, a test filament was produced using the PLA10 blend, identified as having optimal thermo-mechanical and processing characteristics for 3D printing at 148 °C and 8 bar. The filament maintained a diameter of 1.72 ± 0.01 mm, ensuring consistent average ovality throughout the extrusion process.

### 3.3. Cell Viability Assay

**Figure 9:**
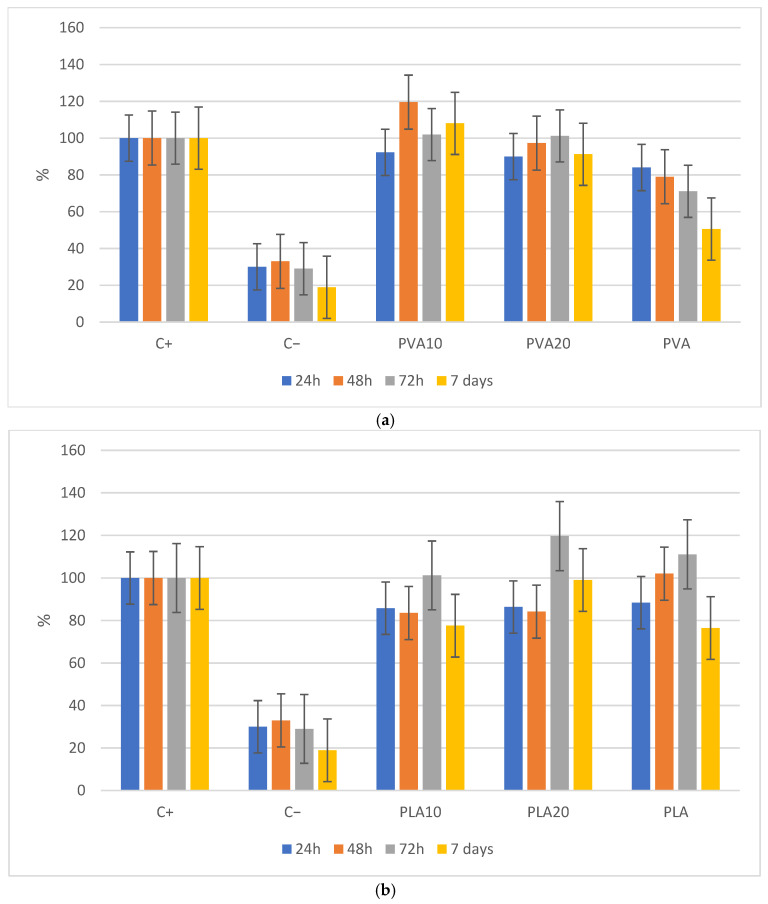
Effect of BCF concentration on cell viability of PVA (**a**) and PLA (**b**) at different time points.

Figure 9 illustrates the effect of varying BCF concentrations on the cell viability of PVA (Figure 9a) and PLA (Figure 9b) over different time intervals. For PVA, the composite containing 10% BCF exhibited the highest cell viability, reaching a peak at 7 days, suggesting this concentration as optimal for enhancing biocompatibility. However, at 20% BCF, viability decreases, although it remains higher than that of pure PVA, indicating a stabilization of the beneficial effect beyond this threshold. Pure PVA shows a progressive decline in viability over time, consistent with its low cell adhesion properties.

In PLA, cell viability increases at 24 h with rising BCF content, peaking at 20%. At 48 h, a decrease is observed compared to pure PLA, suggesting a potential initial adverse effect. By 72 h, viability increases again, with the PLA20 sample reaching the highest value, indicating that ceramic incorporation may promote long-term cell proliferation. After 7 days, viability decreases in the PLA10 composite, whereas the PLA20 sample maintains significantly higher viability, suggesting that higher ceramic content favors cell adhesion over time.

Analysis of pure polymers (0% BCF) reveals that PLA viability increases until 72 h before declining due to nutrient depletion, while PVA peaks at 24 h followed by a progressive decline until day 7. In both cases, the materials do not exhibit cytotoxic effects, as viability initially increases to a maximum. Overall, BCF incorporation positively influences cell viability in both polymers, with the most significant enhancement at 20% for PLA and 10% for PVA. These differences may be attributed to experimental protocol variations: PLA samples were in direct contact with cell cultures, whereas PVA samples involved dilutions in the culture medium.

### 3.4. FEM Simulation

**Figure 10:**
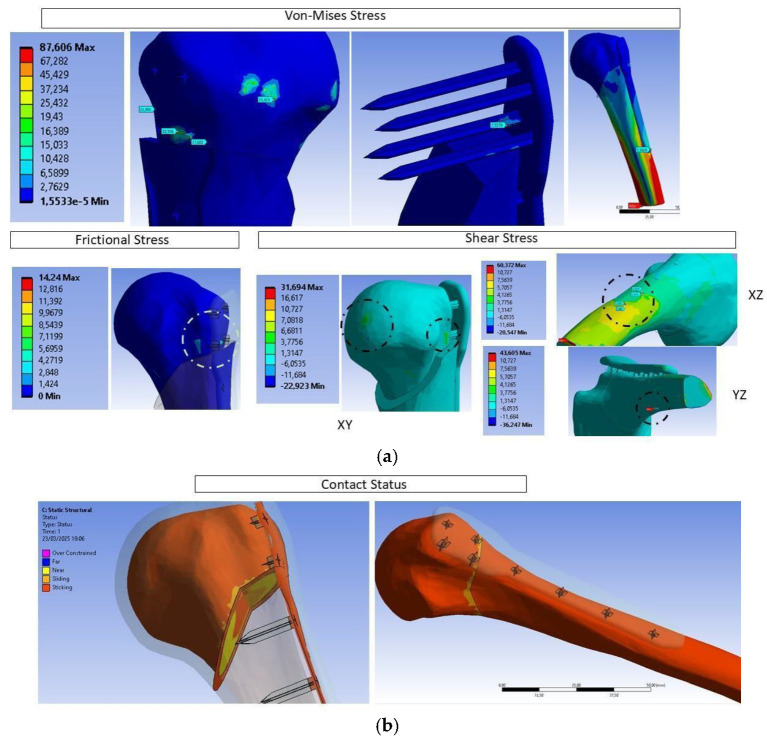
Results obtained under various load conditions at the interfaces between solid components: (**a**) von Mises stress, frictional stress, and shear stress (all in MPa); (**b**) penetration displacement (mm).

The contact area between the proximal plate and the humerus experiences the highest stress and is most susceptible to separation due to insufficient adhesion, which fails to withstand the maximum shear stress at the fracture site. To ensure plate stability, the minimum required adhesion surface in this area must be determined. Figure 10 highlights the fracture zone, where the humerus splits, as the region with the greatest displacement. To identify the critical shear stress responsible for sliding, Coulomb’s friction model was applied [69].

The analysis reveals that certain areas, excluding the force application zone, experience elevated stresses—up to 27 MPa in the cancellous bone and around the holes for plate spikes—while the cortical bone endures stresses of 114 MPa. These stress levels may induce localized yielding; however, they remain below the elastic limit of the PLA10 biomaterial used for the plate, which is approximately 54 MPa. Frictional stress concentrates at the posterolateral junction between the trabecula and the plate, with shear stresses ranging between 10 and 15 MPa. The maximum shear stress occurs on a plane inclined approximately 40° relative to the plane of the applied force. Although fixation of the distal humerus increases the moment in that region, the plate’s adhesion remains stable. These results validate the use of PLA10 as a suitable material capable of withstanding the physiological loads generated by natural shoulder movements. Despite the scarcity of previous studies on non-metallic osteosynthesis plates, it is confirmed that the design retains adhesion even when using spikes in place of metallic screws.

**Figure 11:**
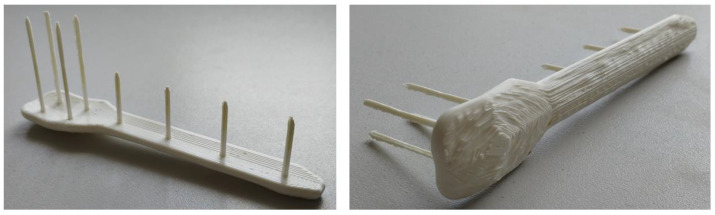
Prototypes printed using 3D filament based on the PLA10 composite.

Figure 11 shows a prototype printed in PLA10 using the same 3D printer (Anycubic Kobra 2 Max) and printing parameters as those employed for the test specimens. The prototypes exhibit morphological irregularities attributable to layer height and the distinctive characteristics of the composite; however, structural stability is maintained throughout the geometry except at the sharp tips of the spikes, where a flawless surface finish was not achieved. Achieving defect-free printing of filaments based on novel composites is particularly challenging and necessitates a dedicated research methodology, which should be pursued complementarily to the objectives of this work.

## 4. Conclusions

Proximal humerus fractures, particularly in elderly patients and veterinary cases, pose challenges due to the limitations of traditional metal plates, such as corrosion and mechanical incompatibility. Consequently, the development of new osteosynthesis plates composed of non-metallic, biocompatible materials that can be customized via technologies like 3D printing is essential. This study demonstrates that patient-specific osteosynthesis plate design—achieved through the multidisciplinary integration of biomedical engineering, biotechnology, materials chemistry, and trauma surgery—enables optimal anatomical adaptation and biomechanical functionality for proximal humerus fractures. The combination of advanced methods such as 3D modeling based on computed tomography, finite element analysis, and additive manufacturing ensures optimal stress distribution and minimizes the risk of mechanical failure, overcoming the limitations of traditional standardized implants.

Our collaborative approach facilitated the development of biocomposites (PLA/BCF and PVA/BCF) wherein the synergy between the polymer matrix and hydroxyapatite derived from agricultural waste simultaneously enhances osteoconductivity, controlled biodegradation, and mechanical properties. Of particular note is the stability of PLA/BCF under humid conditions, making it preferable over PVA/BCF despite lower elasticity, due to its resistance to solubility and maintenance of structural integrity during bone regeneration.

Importantly, this work successfully fabricated biocomposites by blending PLA with up to 20% BCF, a significant milestone scarcely documented in materials engineering literature, demonstrating the feasibility of achieving high ceramic loadings without compromising processability or mechanical performance. Clinical validation of these customized designs, supported by computational simulations and in vitro assays, confirms their potential to reduce postoperative complications such as plate loosening or bone resorption, establishing a new paradigm in sustainable orthopedic implant manufacturing.

Furthermore, the successful 3D printing of prototypes using the PLA10 composite, despite challenges in achieving perfect surface finish at the spike tips due to composite-specific characteristics, confirms the feasibility of producing patient-specific implants with complex geometries. An alternative solution to overcome printing defects at slender spike tips is to print plates and spikes separately and join them using medical-grade epoxy adhesive. This underscores the need for continued research into optimized printing methodologies tailored to novel biocomposites, complementing the objectives of this study and advancing sustainable, customizable osteosynthesis solutions.

## Figures and Tables

**Table 1:** Key Physical and Mechanical Properties of PVA and PLA Filaments According to Ultimaker B.V Manufacturer Data.

PHYSICAL AND MECHANICAL PROPERTIES	PVA	PLA
Density (g/cm^3^)	1.23	1.24
Vicat softening temperature at 10 N (°C)	60.2	59
Tensile Modulus (MPa)	3860	4635.7
Tensile Strength (MPa)	78	55.5
Elongation at break (%)	9.9	1.1
Resistance to Charpy impact test at 23 °C (kJ/m^2^)	1.6	7

**Table 2:** Main properties of BCF biocomposite according to reference [37].

Hydroxyapatite, Ca_10_ (PO_4_)_6_(OH)_2_, wt %	75.0 ± 0.5
β-Tricalcium phosphate (TCP)	25.0 ± 0.5
Ca/P ratio	1.45
pH	7.0 ± 0.5
BET surface areas, m^2^/g	32
Specific gravity, g/cm^3^	2.69 ± 0.005
Particle size, nm	<50
Shelf life, years	2
Physical appearance	white suspension

**Table 4:** Crystallization rates of pure polymers and their biocomposites.

Samples	T_m_ [°C]	ΔH_c_ [J/g]	χ_c_ [%]
pure PLA [48,49]		91–93	100
Processed pure PLA	151.20	10.45	11.48
PLA10	151.06	10.15	13.94
PLA20	150.99	4.94	7.76
pure PVA [50,62]		156	100
Processed pure PVA	178	14.6	8.85
PVA10	177.87	13.24	10.03
PVA20	177.19	10.48	9.07

## Data Availability

The original contributions presented in this study are included in the article. Further inquiries can be directed to the corresponding author.

## Nomenclature

BCF	BBCFiller^®^
BET	Brunauer–Emmett–Teller surface area (m^2^/g)
CAD	Computer-Aided Design
CT	Computed Tomography
D	Apparent density (kg/m^3^)
DICOM	Digital Imaging and Communication in Medicine
DSC	Differential Scanning Calorimetry
E	Young’s modulus (MPa)
EDX/EDS	Energy Dispersive X-ray Spectroscopy
ε_b_	Elongation at maximum strain (%)
FEM	Finite Element Method
FFF	Fused Filament Fabrication
FTIR	Fourier Transform Infrared Spectroscopy
GCI	Grid Convergence Index
HA	Hydroxyapatite
Hcpolymer	Crystallinity enthalpy of the pure polymer (100% crystallinity)
Hcbiocomp	Crystallinity enthalpy of the biocomposite
hFOB	Human fetal osteoblastic cells
J_c_	Impact strength (kJ/m^2^)
MAH	Maleic anhydride
MFI	Melt Flow Index (g/10 min)
PLA	Polylactic acid
PLA10	PLA with 10% BCF
PLA20	PLA with 20% BCF
PLA103D	3D-printed PLA10
PVA	Polyvinyl alcohol
PVA10	PVA with 10% BCF
PVA20	PVA with 20% BCF
SEM	Scanning Electron Microscopy
σ_b_	Stress at break (MPa)
TCP	Tricalcium phosphate (general term; see β-TCP)
T_m_	Melting temperature (°C)
Wpolymer	Polymer mass fraction in the biocomposite
XRD	X-ray Diffraction
β-TCP	Beta-tricalcium phosphate
ΔH	Crystallization enthalpy (J/g)
χ_c_	Degree of crystallinity (%)
